# Chimeric Antigen Receptor Based Cellular Therapy for Treatment Of T-Cell Malignancies

**DOI:** 10.3389/fonc.2022.876758

**Published:** 2022-05-06

**Authors:** Kamila Polgárová, Pavel Otáhal, Cyril Šálek, Robert Pytlík

**Affiliations:** ^1^ 1st Department of Medicine, First Faculty of Medicine, Charles University, Prague, Czechia; ^2^ 1^st^ Department of Medicine, General University Hospital in Prague, Prague, Czechia; ^3^ Department of Immunotherapy, Institute of Haematology and Blood Transfusion, Prague, Czechia; ^4^ Institute of Clinical and Experimental Hematology, First Faculty of Medicine, Charles University, Prague, Czechia; ^5^ Clinical Department, Institute of Haematology and Blood Transfusion, Prague, Czechia; ^6^ Department of Cell Therapy, Institute of Haematology and Blood Transfusion, Prague, Czechia

**Keywords:** T-cell lymphoma, T-cell acute lymphoblastic leukemia/lymphoblastic lymphoma, therapy, immunotherapy, chimeric antigen receptor (CAR), CAR-T cells

## Abstract

T-cell malignancies can be divided into precursor (T-acute lymphoblastic leukemia/lymphoblastic lymphoma, T-ALL/LBL) and mature T-cell neoplasms, which are comprised of 28 different entities. Most of these malignancies are aggressive with rather poor prognosis. Prognosis of relapsed/refractory (R/R) disease is especially dismal, with an expected survival only several months after progression. Targeted therapies, such as antiCD30 immunotoxin brentuximab vedotin, antiCD38 antibody daratumumab, and anti-CCR4 antibody mogamulizumab are effective only in subsets of patients with T-cell neoplasms. T-cells equipped with chimeric antigen receptor (CAR-Ts) are routinely used for treatment of R/R B-cell malignancies, however, there are specific obstacles for their use in T-cell leukemias and lymphomas which are fratricide killing, risk of transfection of malignant cells, and T-cell aplasia. The solution for these problems relies on target antigen selection, CRISPR/Cas9 or TALEN gene editing, posttranslational regulation of CAR-T surface antigen expression, and safety switches. Structural chromosomal changes and global changes in gene expression were observed with gene-edited products. We identified 49 studies of CAR-based therapies registered on www.clinicaltrials.gov. Most of them target CD30 or CD7 antigen. Results are available only for a minority of these studies. In general, clinical responses are above 50% but reported follow-up is very short. Specific toxicities of CAR-based therapies, namely cytokine release syndrome (CRS), seem to be connected with the antigen of interest and source of cells for manufacturing. CRS is more frequent in antiCD7 CAR-T cells than in antiCD30 cells, but it is mild in most patients. More severe CRS was observed after gene-edited allogeneic CAR-T cells. Immune effector cell associated neurotoxicity (ICANS) was mild and infrequent. Graft-versus-host disease (GvHD) after allogeneic CAR-T cells from previous hematopoietic stem cell donor was also observed. Most frequent toxicities, similarly to antiCD19 CAR-T cells, are cytopenias. CAR-based cellular therapy seems feasible and effective for T-cell malignancies, however, the optimal design of CAR-based products is still unknown and long-term follow-up is needed for evaluation of their true potential.

## T Cell Non-Hodgkin’s Lymphomas

T-cell non-Hodgkin’s lymphomas (T-NHL) represent a heterogeneous group of uncommon malignancies comprising approximately 10-15% of all non-Hodgkin’s lymphomas with variable, geographically-dependent incidence (1). They are comprised of precursor T-cell neoplasms, peripheral systemic nodal and extranodal T-NHL (PTCL), and primarily cutaneous T-cell lymphomas (CTCL).

The only recognized precursor T-cell neoplasms in recent WHO classification are T-lymphoblastic leukemia/lymphoma (T-ALL/LBL) and early T-cell precursor lymphoblastic leukemia (ETP-ALL). T-cell acute lymphoblastic leukemia (ALL) is an aggressive malignancy that accounts for 25% of adult ALL cases with long-term survival of 50–60% in the adult population (2). The prognosis of ALL in adults in general has considerably improved during past decades. This was mainly due to the introduction of intensive pediatric-based protocols and the advent of novel targeted agents. However, immunotherapeutic drugs approved for clinical use are limited almost exclusively to the B-cell precursor ALL. On the contrary, therapeutic approach to the T-ALL remains unchanged for decades. The last drug that has been developed for treatment of T-ALL was nelarabine in 2005 (3–5). The absence of new treatment options turns the prognosis dismal in resistant or relapsed disease. This is the issue mainly in subjects with early or mature T-cell phenotypes which are considered high risk in most national treatment protocols and are referred for allogeneic stem cell transplantation (alloHSCT) in the first complete remission (6).

Early precursor T-ALL (ETP-ALL) is an entity with a distinct immunophenotype and gene expression profile which is distinguished by chemotherapy resistance, slow MRD response, and more frequent relapse (7–9). The adverse outcome of ETP-ALL could be overcome by risk-adapted treatment intensification and the inclusion of new agents.

The prognosis of relapsed T-ALL is critical. Only 30% of patients with early relapse and 42% with late relapse achieve a second complete remission. Consolidation with alloHSCT appears to be the only treatment eventuality with curative potential in relapsed adult ALL with a 3-year overall survival of 38%. Median survival without alloHSCT is only 2.7 months (10).

Treatment of relapsed and refractory (R/R) T-ALL presents an unmet medical need. There is an urgent demand to develop innovative treatment approaches that take into consideration recent discoveries in disease biology and molecular mechanisms. Combination therapy with venetoclax and chemotherapy has shown promising efficacy in R/R T-ALL (11). Dual blockade of BCL-2/BCL-XL/BCL-W with venetoclax and navitoclax is hoped to improve efficacy (studies **NCT03181126, NCT05054465**).

Leukemic blasts in T-ALL have robust surface CD38 expression that remains stable after exposure to multiagent chemotherapy. This justifies for concurrent administration of daratumumab and chemotherapy which has been tested in R/R T-ALL (12–14). A clinical study with daratumumab and chemotherapy is underway (**NCT03384654**). Other biologicals tested in T-ALL include the antiCD30 immunoconjugate brentuximab vedotin (**NCT03264131, NCT02588651, NCT03113500**), the BCL-2 inhibitor venetoclax in combination with hypomethylating agent azacitidine (**NCT05149378**), the PI3K inhibitor duvelisib in combination with histone deacetylase inhibitor (HDACi) romidepsin and azacitidine (**NCT04639843**), mTOR inhibitor everolimus in combination with chemotherapy (**NCT03328104**), dual inhibitor of EZH1/2 methyltransferase (**NCT04102150**), or anti-IL-2R immunotoxin LMB-2 in combination with immunosuppressive chemotherapy (**NCT00924170**). However, despite the promises that these treatments bring, new strategies need to be investigated.

For mature NK/T-cell neoplasms (both systemic and cutaneous), the recent WHO classification recognize 28 distinct clinicopathological entities. A list of them, together with their five-year prognosis with current treatment options, is show in **Table 1**.

**Table 1:** Outcomes of selected types of mature T-cell and NK-cell neoplasms (according to WHO 2016 classification).

Mature T-cell and NK-cell neoplasm based on WHO 2016 classification	5yrs OS	Ref
T-cell prolymphocytic leukemia	10%	(15)
T-cell large granular lymphocytic leukemia	65%	(16)
Chronic lymphoproliferative disorder of NK cells		
Aggressive NK-cell leukemia	0%	(17)
Systemic EBV^+^ T-cell lymphoma of childhood		
Hydroa vacciniforme–like lymphoproliferative disorder	38%	(18)
Adult T-cell leukemia/lymphoma	7%*- 28%**	(1)
Extranodal NK-/T-cell lymphoma, nasal type	0%*- 57%**	(1)
Enteropathy-associated T-cell lymphoma	14%*- 29%**	(1)
Monomorphic epitheliotropic intestinal T-cell lymphoma		
Indolent T-cell lymphoproliferative disorder of the GI tract		
Hepatosplenic T-cell lymphoma	0%	(1)
Subcutaneous panniculitis-like T-cell lymphoma	0%*- 60%**	(1)
Mycosis fungoides	20% - 90%	(19)
Sézary syndrome	26%	(19)
Primary cutaneous CD30^+^ T-cell lymphoproliferative disorders		
Lymphomatoid papulosis	92	(20)
Primary cutaneous anaplastic large cell lymphoma	100%*	(1)
Primary cutaneous γδ T-cell lymphoma	11	(21)
Primary cutaneous CD8^+^ aggressive epidermotropic cytotoxic T-cell lymphoma	32	(22)
Primary cutaneous acral CD8^+^ T-cell lymphoma		
Primary cutaneous CD4^+^ small/medium T-cell lymphoproliferative disorder		
Peripheral T-cell lymphoma, NOS	11%*- 50%**	(1)
Angioimmunoblastic T-cell lymphoma	25%*- 56%**	(1)
Follicular T-cell lymphoma		
Nodal peripheral T-cell lymphoma with TFH phenotype	50%	(23)
Anaplastic large-cell lymphoma, ALK^+^	33%*- 90%**	(1)
Anaplastic large-cell lymphoma, ALK^−^	13%*- 74%**	(1)
Breast implant–associated anaplastic large-cell lymphoma	75% - 98%	(1)

*IPI (international prognostic index for aggressive lymphomas) 0-1, **IPI 4-5.

The most frequent subtypes, comprising 60% of all T-NHL cases, are peripheral T-cell lymphoma not otherwise specified (PTCL-NOS), angioimmunoblastic T-cell lymphoma (AITL), and systemic anaplastic large cell lymphoma (sALCL) (24). With the exception of ALK+ sALCL, where 5-year overall survival (OS) is 90% with low-risk features, the common feature of most systemic PTCL is aggressive course and poor treatment response as shown by the International T-cell lymphoma project (**Table 1**) (1). In recent years, several major advances in understanding T-NHL have been made, including the description of recurrent chromosomal and molecular aberrancies and disruptions in several signaling pathways (25). Despite these discoveries, therapeutic improvement over last two decades has been disappointing and has not altered the dramatically poor prognosis of PTCL patients.

Established first-line treatment options usually include an anthracycline-based regimen, such as CHOP (cyclophosphamide, vincristine, doxorubicin, prednisolone), as extrapolated from the treatment strategy of aggressive B-NHL. Although there are some controversies in terms of etoposide use in different patient populations (26), younger patients may benefit from its addition to CHOP (27).

Several other attempts to improve the CHOP “backbone” have been made, including adding denileukin-diftitox, alemtuzumab, or romidepsin. However, most of these efforts failed to improve OS and/or added unacceptable toxicity and thus were not incorporated into treatment routine (28–30). The first study proving survival benefit without significantly increasing toxicity when adding a new agent to the CHOP-backbone was ECHELON-2 (31). In this randomized prospective trial, an antiCD30 antibody-drug conjugate brentuximab-vedotin (BV) was combined with CHP (without vincristine) and compared to CHOP in first-line treatment of patients with PTCL. In ALCL subcohort, CHP-BV proved to be superior to CHOP with regards to both event-free survival (EFS) and overall survival (OS). No benefit was found in CD30 positive PTCL-NOS or AITL, though this study was not powered enough to detect statistical differences in these populations. New first line combinations, such as azacitidine or duvelisib with CHO(E)P (**NCT03542266, NCT04803201**), are studied, yet the data are too immature to predict their further impact on front-line treatment approach.

Autologous stem cell transplant (ASCT) has often been offered as a consolidation in upfront settings, although, yet again, its significance has been both questioned and highlighted by different reports (32). Currently, no randomized trials are available. Allogeneic hematopoietic stem cell transplant did not bring any survival benefit in consolidation settings after first remission achievement, which, considering high treatment-related mortality (TRM) eliminated this approach from front-line settings (33, 34).

More than 50% of patients with PTCL, including those treated with BV, develop relapse or disease progression after first line therapy. According to several reports, median OS in R/R setting is 3.0-5.5 months. More favorable outcomes are observed in patients with good performance status and those treated by chemotherapy and stem cell transplantation (SCT); histological subtype, except for ALK+ sALCL, does not affect the prognosis (35, 36).

In patients with R/R disease, standard of care is not defined. Different scenarios may include conventional chemotherapy with or without SCT, antibody-based therapy, and so called “targeted therapy” (such as HDACi, hypomethylating agents, PI3Ki or immune modifiers alone or in combinations). Second line chemotherapies mostly employ platinum derivative regimen or gemcitabine as a single agent or in combination with corticosteroids and platinum derivative. No significant differences between different therapeutic approaches in achieving anti-lymphoma response or its duration in R/R PTCL were reported (37, 38).

In the intent-to-cure setting, consolidation by SCT might be discussed. Prospective randomized trials are lacking, however, expert consensus recommends ASCT for relapsed chemosensitive disease, when it was not performed frontline and consideration of alloHSCT in some cases. Conversely, several studies showed disappointing results after ASCT in R/R settings with high relapse rates of 50-70% within the first year, except for sALCL, where the results were more promising (38–40). AlloHSCT may be an option for long term disease control. As shown by several retrospective reports, the 3yrs OS and PFS reaches 81% and 64%, respectively with TRM 12-31% in different trials. However, considering the median age of diagnosis in PTCL patients, aggressive disease course with more than 60% of patients not achieving at least PR, and toxicity of previous therapies, only minority of patients are eligible for alloHSCT (41–43).

Targeted therapy (comprehensively listed in **Supplementary Table 1**) did not fulfill the hopes for better responses either. Brentuximab vedotin, already mentioned in first-line setting, had impressive results when used for salvage therapy in ALCL (44) and as shown by the ECHELON-2 study, this agent is active also in retreatment of patients who received it frontline. However, most PTCL are CD30 negative. Other approaches currently approved by FDA and/or Chinese and Japanese authorities for R/R PTCL treatment include pralatrexate and HDACi such as vorinostat, romidepsin, belinostat, and chidamide. These agents lead to response in 5-40% of patients (37, 45, 46). The approval of these drugs by EMA, however, was refused, as most trials did not prove a survival benefit in R/R PTCL population. Lenalidomide was also tested, as a single agent as well as combined with HDACi and corticosteroids, but the result were unsatisfying, with ORR 25-30% and PFS 2.2-2.5 months (47, 48). The use of mogamulizumab (antiCCR4 monoclonal antibody) in R/R PTCL was similarly disappointing and the agent is currently used only for R/R CTCL. Alemtuzumab (antiCD52 monoclonal antibody), used in prolymphocytic leukemia and other T-cell malignancies, did not bring better results for patients with R/R PTCL either, and led to unacceptable toxicity (49). Other investigated agents include ALK-inhibitors for ALK+ ALCL (crizotinib is already approved for treatment of -children and young adults); PI3K inhibitors, such as duvelisib (**NCT03372057**), hypomethylating agents – azacytidine or decitabine (**NCT04480099, NCT04747236**) or immune checkpoint inhibitors (**NCT04795869, NCT03278782**).

Overall, the prognosis of patients with T-NHL remains unfavorable. Even with all currently available therapeutic strategies, up to 45% of PTCL patients present as primarily refractory to the first line treatment and the other 15% relapse in ≤ 12 months (1, 24) with median survival of only few months. Thus, new approaches are urgently needed. Treatment with immune cells equipped with chimeric antigen receptors (CAR) seems promising, though it presents specific challenges in patients with T-cell malignancies.

## Basic Concepts and Challenges in Chimeric Antigen Receptor-Based Therapy of T-Cell Malignancies

Chimeric antigen receptor (CAR)-based therapy is a unique treatment method that includes living cells and similarly to other cellular products, it is classified as advanced therapy medicinal product (ATMP). From the regulative perspective, however, the production is covered by Pharmaceutical Acts, similarly to other treatment agents (50). Though first reports about successful construction of fusion receptors were published in 1991, it took more than 25 years for the approval of the first CAR-based therapy directed against R/R CD19 lymphoid malignancies (51). Products available in Europe may be used for treatment of aggressive B-cell lymphomas (51–53), B-ALL in children and young adults (54), and mantle cell lymphoma (55); and approval for follicular lymphoma treatment is pending. BCMA-directed treatment for multi-resistant multiple myeloma is also available (56). All these products are apparently successful in cases resistant to chemo(immuno)therapy and even in some patients with active and progressing disease at the time of infusion. Though universal, off-the shelf allogeneic products are in development (see below), and all currently registered CAR-T products are based on autologous T-lymphocytes. Engraftment and further expansion of CAR products after infusion is facilitated by lymphodepletion chemotherapy, mainly based on fludarabine with cyclophosphamide and/or bendamustine. These treatments may have some uncommon toxicities, namely cytokine release syndrome (CRS), a combination of fever, hypoxia, hypotension, and capillary leak syndrome, with or without other organ manifestations (renal failure, coagulopathy, or neurological symptoms). Laboratory findings include elevated levels of CRP, IL-1β, IL-2, IL-6, IL-10, IFN-γ, and TNF-α. On a pathophysiological level, CRS seems to be mediated by T-lymphocytes and myeloid cells interactions. CRS usually starts 1-3 days after CAR-T infusion and abates in one week, though late onsets and protracted courses may occur. Treatment includes antibodies against IL-6 or against IL-6 receptor and corticosteroids (57). Immune effector cell associated neurotoxicity syndrome (ICANS) is presented by several symptoms, such as confusion, aphasia, apraxia, frontal release sings, meningism, palsy, seizures, cerebral edema, or coma (58). ICANS usually occurs later than CRS, though there seems to be a connection between CRS severity and ICANS occurrence. Similar to CRS, it is probably induced by the inflammatory cascade triggered by activation of T-lymphocytes and their interaction with myeloid cells. Inflammation-induced endothelial dysfunction was also implied in ICANS pathogenesis (59). Most of the ICANS symptoms resolve within a few weeks, but irreversible or even fatal toxicities have been reported. Corticosteroids are used for inflammatory cascade control but anti-IL-6 therapy, used to treat CRS does not seem to limit ICANS symptoms. It should be also noted that, in contrast to CAR-T, CAR-NK, treatment is only infrequently associated with CRS or ICANS (60).

## CAR-Based Therapy Against T-Cell Malignancies – Pitfalls and Overcoming Strategies

**Figure 1:**
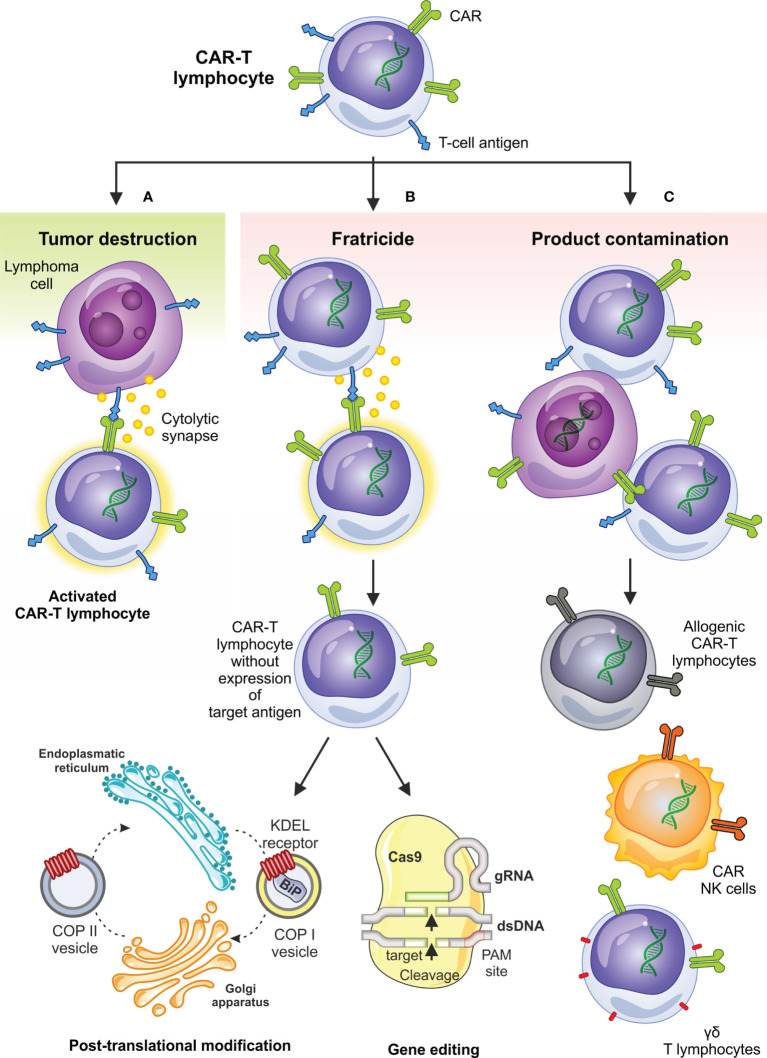
Desired and undesired effects of CAR-based therapy in T-cell malignancies and selected circumventing approaches. Besides on-target antitumor activity **(A)** the anti-T-NHL CAR-T cell therapy can be complicated by fratricide – on target killing of CAR-T cells expressing T-cell antigens **(B)**, which can be overcome by limiting superficial T-cell antigen expression using genetic as well as posttranslational modifications; contamination of the final product by transduced tumor cells **(C)** is another undesired effect observed in anti-T-NHL CAR-T setting and can be overcome by the use of allogeneic CAR-T cells, transduction of NK-cells or by selection and use of ƴδ T lymphocytes. BiP, immunoglobulin heavy chain binding protein; COPI, coated protein I; COPII, coated protein II; ds-DNA, double-stranded DNA; gRNA, guiding RNA; KDEL receptor, K = lysine, D = aspartic acid, E = glutamic acid, L = leucin; PAM, protospacer adjacent motif.

In addition to desired anti-tumor effects, CAR-based therapy for T-cell malignancies also has several pitfalls which may not be an issue in treatment of B- or plasma-cell malignancies. These are mainly fratricide, i.e., on-target killing of all cells expressing the antigen including CAR-T cells, product contamination with transducted tumor cells, and long-term T-cell aplasia **(** **Figure 1** **).**

### Antigen Selection and Fratricide

The essential problem of CAR-T based therapy of T-cell malignancies is the lack of suitable and sufficiently selective target antigen. Most of the antigens of interest by CAR-T cells are expressed by normal T cells as well, which include CD4, CD5, CD7, CD30, and TCR-beta chain. Targeting of these antigens leads to the fratricide, or mutual killing of CAR-T cells during expansion, as these antigens are also expressed by the CAR-T cells.

Interestingly, CD5, a pan-T antigen induces only limited fratricide despite its expression by CAR-T cells and does not impair their expansion. This phenomenon results from the rapid downregulation of the surface expression of CD5 due to rapid internalization upon binding to a specific antibody (61). CD5, however, is not a universal target because it is not expressed sufficiently by many T-cell tumors. Another interesting target limiting the fratricide problem is the T-cell receptor beta-chain (62). The mutually exclusive expression of T-cell receptor β-chain constant domains 1 and 2 (TRBC1 and TRBC2) enables effective targeting of a subset of T-cell malignancies because normal T cell population is composed of both TRBC1 and TRBC2 subsets, while malignancies are clonal and positive for only one T-cell receptor beta chain constant domain. The development of CAR-T cells specific to each one of them enables to select the cancer-specific subset. CD4 is also expressed by only a subset of T cells (63). However, while targeting CD4 partially prevents fratricide during the production, the eradication of normal CD4+ T cells would result in T-cell aplasia and subsequently in HIV/AIDS-like syndrome. Campath-1H, an antiCD52 antibody which depletes CAR-T cells after treatment, may be used as a safety switch (64), however, it also depletes the rest of normal T-cells rather non-selectively. Nevertheless, the CD4-specific CAR-T cells are currently investigated in clinical trials. One of the most promising targets for the therapy of T-cell malignancies by CAR-T cells is CD30, also known as TNFRSF8 (65). On the contrary to CD7 or CD4, it is expressed only on a small subset of normal T-cells but it is expressed in various T-cell malignancies including Hodgkin’s lymphoma (HL), T-ALL, ALCL, AITL, and PTCL-NOS. As discussed earlier, the success of antiCD30 immunotoxin brentuximab vedotin in these diseases has proven that CD30 antigen is a relevant target for CAR-T therapy (see below).

Several other strategies to circumvent fratricide were tested and, in general, rely on targeted removal of CAR-specific T-cell antigen from CAR-T cells by gene-editing approaches based on DNA nucleases CRISPR/Cas9, or TALEN (66), or posttranslational processing (67). Deletion of CD7 and the T-cell receptor alpha chain (TRAC) using CRISPR/Cas9 and transduction of these same allogeneic T cells with a CD7-specific CAR enabled efficient targeting and killing of malignant T cells without significant fratricide of CAR-T cells. TRAC deletion blocked TCR mediated signaling, thus limiting life-threatening GvHD and permitting the safe use of allogeneic T cells as the source of CAR-T cells. Gomes-Silva and colleagues used the CRISPR-Cas9 approach to disrupt the antigen CD7 in the T cells used for the generation of CD7-specific CAR-T cells (68). Their preclinical study showed that targeted genomic disruption of the CD7 gene rendered CD7 CAR T cells resistant to fratricide, permitting robust expansion without compromising T-cell antigen recognition through their native or chimeric receptors. Similarly, Cooper et al. generated using CRISPR-Cas9 a CD7-specific CAR-T cells with disrupted expression of CD7 and TCR alpha chain (69) and produced universal CD7 CAR T cells (UCART7). These UCART7 cells efficiently killed human T-ALL cell lines and patient-derived primary T-ALL *in vitro* and *in vivo* without resulting in xenogeneic GvHD (70). Conversely, it should be mentioned that excessive genome editing can also induce unwanted chromosomal changes, as will be discussed below. Selected antigens examined for use as CAR-T cell targets in T-cell malignancies together with advantages and disadvantages of their use are summarized in **Table 2**. Finally, one of the strategies to overcome the fratricide issue is to use natural killer (NK) cells expressing T-cell targeted CAR.

**Table 2:** Selected antigens used to target T-cell malignancies with the main dis/advantages of their use.

	Normal tissue expression	T-cell malignancies expression (% frequency)	Advantages	Disadvantages	Ref
**Pan-T-cell antigens**					
CD2	T lymphocytes	PTCL-NOS (70%) AITL (100%)	extended expression on T-NHL	fratricide; T-cell depletion	(71, 72)
CD3	T lymphocytes	T-ALL (33%), PTCL-NOS (60%), AITL (70%), ALCL (32%), ATLL (80%)	Extended expression on T-NHL	fratricide; T-cell depletion	(72)
CD5	thymocytes, T lymphocytes	T-ALL (90%), PTCL-NOS (85%), AITL (96%), ALCL (30%), ATLL (85%)	resistance to fratricide; limited toxicity against non-malignant T cells	Fratricide and CAR-T exhaustion (although in lower levels)	(61, 72, 73)
CD7	thymocytes, T lymphocytes, NK cells	T-ALL (95%), PTCL-NOS (50%), AITL (57%), ALCL (32-54%), ATLL (25%)	extended expression on T-cell malignancies	fulminant fratricide precluding expansion; T and NK cell depletion	(72, 73)
**Antigens with restricted expression**					
CD4	CD4^+^ T cells, monocytes, dendritic cells	T-ALL (10%), PTCL-NOS (60%), AITL (86%), ALCL (63%), ATLL (94%)	extended expression on T-cell malignancies	CD4^+^ T-cell depletion with AIDS-like syndrome	(63, 72)
CD30	subsets of B and T cells	PTCL-NOS (16%), ALCL (93%), AITL (32-50%), ATLL (39%)	selectivity and absence of fratricide risk	present only on subset of T-cell malignancies	(74)
TRBC1 TRBC2	T lymphocytes	PTCL-NOS (27%), ALCL (25%), AITL (34%)	selectivity and absence of fratricide risk	limited CAR-T persistence due to crosslinking with TCR on healthy T cells	(62, 75)
CCR4	T cell subsets, basophils, megakaryocytes	T-ALL (0%), PTCL (34%), ATLL (88%)	selectivity	Disappointing results for PTCL in mAb use; on target of tumor toxicity	(72, 76)
CD70	Activated and mucosal T cells; (pre) inflammated gastrointestinal mucosa	PTCL-NOS (64%), ALCL (50-78%), CTCL (95%)	high expression on T-lymphoma cells; low risk of on-target/off-tumor toxicity shown in xenografts models	possible on-target off-tumor toxicity	(77, 78)
CD147	pleiotropic expression (CNS, GIT, thymocytes)	ALCL ALK^-^ (28%), ALCL ALK^+^ (89%),	strong expression in ALK^+^ ALCL	possible on-target off-tumor toxicity	(79, 80)

AITL, angioimmunoblastic T-cell lymphoma; ALCL, anaplastic large cell lymphoma; ALK, anaplastic lymphoma kinase; ALL, acute lymphoblastic leukemia; ATLL, adult T-cell leukemia/lymphoma; CCR4, C-C chemokine receptor type 4; CNS, central nervous system; CTCL, cutaneous T-cell lymphoma; GIT, gastrointestinal tract; NHL, non-Hodgkin’s lymphoma; NK, natural killer; NOS, not otherwise specified; PTCL, peripheral T-cell lymphoma; TCR, T-cell receptor; TRBC, T-cell receptor β-chain constant domain.

### Contamination of the Product by Malignant Cells

The manufacturing of CAR-T cells is further complicated by the frequent presence of malignant T cells in the peripheral blood which can thus lead to their transduction with the viral vector during manufacturing and contamination of the produced CAR-T cells. Using healthy donors for CAR-T cells production efficiently eliminates the risk of malignant cell contamination. The use of allogeneic CAR-T cells, however, is rendered by the elimination of endogenous T-cell receptor, mostly using gene editing techniques to reduce alloreactivity with graft versus host disease risk (see above). Strategies similar to the abovementioned limitation of superficial T-cell antigens expression can be used.

### T-Cell Aplasia

T-cell aplasia is a problem connected not only with antiCD4 CAR equipped cells but also with targeting other pan-T-cell antigens. Unlike B-cell aplasia with antiCD19 CAR-T cells, which can be easily addressed by immunoglobulin substitution, T-cell aplasia has no easy remedy. Various so-called safety switches were developed for targeted elimination of CAR-T cells (81). In fact, the importance of safety switches applies to all types of CAR-T cells since the ability to control the activity of T cells *in vivo* increases the safety of clinical testing in general. Unfortunately, some of the highly effective technologies (as utilizing rimiducid-based inducible caspase 9) cannot be widely used in clinical trials due to limited availability of the pharmaceutical-grade compounds. In contrary, a simple, effective, and clinically validated safety switch can be based on the use of therapeutic antibodies to surface antigens EGFR and CD20. Co-expression of these antigens with CAR construct enables rapid elimination of administered CAR-T cells with therapeutic antibodies cetuximab and rituximab, respectively (82).

### Genetic Manipulation, Oncogenesis Risk

Gene engineering techniques (summarized in **Table 3**) rely on two types of DNA editing: first, the ability to insert a large transgene up to several kilobases long into the genome and second, to disrupt a chosen gene *via* targeted mutagenesis. Such modifications of DNA might result in dysregulation of expression of endogenous genes by strong promoters such as EF1a-alpha used for the expression of therapeutical transgene. Currently used vectors for production of clinical-grade CAR-T cells are lentiviral/retroviral vector systems (LV/RV) and despite the fact that LV/RVs introduce the transgene randomly into the genome, both viral systems have proved to be safe in thousands of patients treated with CD19-specific CAR-T cells. Furthermore, lentiviral vectors, derived from the human immunodeficiency virus, have been extensively optimized over the past two decades and became a gold standard for production of clinical-grade CAR-T cells used in multiple clinical trials. Thus, the theoretical concern of insertional oncogenesis with lentiviral vectors has not been substantiated so far. Unfortunately, production of GMP-grade viral vectors is highly complicated due to extensive regulatory issues. Commercial on-demand production is therefore slow and bears extreme costs. The complicated nature of GMP production of LV/RVs limits the development of novel types of CAR-T cells especially in academic settings (83). To overcome such limitations, non-viral systems utilizing transposons such as Sleeping Beauty (SB) and piggyBac (PB) were developed and are already used in clinical trials (84–86). Both transposon systems insert genes randomly into the genome similarly to viral vectors, although slight differences in the integration pattern were described (87). Engineering *via* transposons requires GMP production only of DNA plasmids compared to complicated packaging of LV/RVs in producer cell lines. Additionally, SB/PB transposons are capable to insert much larger DNA segments than any viral vector. The ability to insert large DNA sequence into the genome might allow the generation of CAR-T enhanced by co-expression of cytokines, or with multiple CAR constructs and safety switches, or containing complex expression cassettes regulating activity of CAR-T. Conversely, the possibility of serious adverse events caused by the random insertion of CAR transgene should not be underestimated. Reports by Bishop et al. described a development of CAR-T-cell lymphoma in 2 of 10 patients effectively treated with piggyBac-modified CD19 CAR-T cells (92). Lymphomas showed a high transgene copy number of the transposon but no insertion into typical oncogenes. Furthermore, structural changes such as altered genomic copy number and point mutations unrelated to the insertion sites were also described. Transcriptome analysis showed that the global changes in transcription predominantly correlated with the gene copy number rather than insertion sites. Also, both patients underwent allogeneic hematopoietic stem cell transplantation prior to CAR-T treatment and received a donor-derived CAR-T cell product, thus, other factors related to this procedure might have contributed to the pathological expansion of administered CAR-T cells (92).

**Table 3:** Summary of different gene editing techniques with the main dis/advantages of their use.

Technology	Advantages	Disadvantages	Ref
Lentiviral/retroviral vectors	no insertional oncogenesis reported after several years of use	complicated and slow manufacturing	(83)
Transposons PiggyBack Sleeping Beauty	easy manufacturing, large inserts allowing insertion of multiple CAR constructs, safety switches or other armamentarium	risk of insertional mutagenesis/chromosomal aberrations, CAR-T cell derived lymphomas reported	(84–87)
Targeted mutagenesis TALEN CRISPR/Cas9	targeted gene editing	increased risk of mutagenesis including chromotripsis	(88–91)

Similar to the insertional oncogenesis caused by random integration of the transgene, targeted mutagenesis of DNA *via* DNA nucleases such as CRISPR/Cas9 or TALEN represents a challenging task to evaluate all possible risks associated with the modification of DNA. On the subcellular level, it was found that the generation of DNA double-strand breaks (DSBs) by CRISPR/Cas9 or TALEN editing can give rise to a broad spectrum of chromosome structural abnormalities such as chromothripsis, a process characterized by large chromosome rearrangements and deletion of large DNA fragments restricted to one or a few chromosomes (88). Gene editing is most frequently used for production of the allogeneic CAR-T cells and is based on the disruption of endogenous TCR alpha/beta chains *via* targeted mutagenesis (89). In addition, disruption of inhibitory receptors such as PD-1 has been clinically tested as well. For example, in a published clinical study (90) authors used CRISPR/Cas9 editing to disrupt the endogenous T cell receptor (TCR) chains alpha and beta to reduce TCR mispairing and removed the gene for PD-1 inhibitory receptor for improvement of antitumor immunity. Next, engineered T cells were transduced with a lentiviral vector expressing a cancer-specific TCR transgene (NY-ESO-1) and used in patients with refractory cancer. This interesting study, however, did not demonstrate any significant positive effect of PD-1 disruption on the *in vivo* survival of CAR-Ts. Moreover, chromosomal translocations were detected in the administered T cells. Despite the fact their frequency decreased over time, these results suggest that large genomic changes might occur. Reports from other clinical trials with allogeneic CAR-T cells such as **NCT04416984** (ALPHA2) showed similar signs of unwanted mutagenesis, resulting from TALEN editing leading to a temporary stop by the FDA (93). Both cases clearly raised the regulatory requirements of quality control tests in any further clinical trials utilizing CRISPR/Cas9 or TALEN technology.

Importantly, unwanted off-target effects of genome editing *via* nucleases resulting from DNA-DSBs have been widely described and can be controlled by optimized guide-RNA (gRNA) and/or highly specific Cas9 nucleases (91). The described cases of unwanted on-target effects, however, lack in-depth exploration and raise major safety issues regarding genome integrity. Thus, both CRISPR/Cas9 and TALEN nucleases can produce major genomic alterations and these findings might limit their use for production of clinical-grade engineered T cells. From the perspective of the regulatory agency, extensive safety and quality control tests of generated cells will be required for approval of new clinical trials with experimental therapeutical products engineered with CRISPR/Cas9, or TALEN. Unexplained safety concerns resulting from the on-target side effects of DNA editing might therefore complicate the approval of novel experimental CAR-T products.

### CAR-NK Cells

NK cells, as a part of innate immune system, use similar killing strategies of malignant or virus-infected cells as cytotoxic T lymphocytes. However, in contrast to T cells with wide repertoire of clonally rearranged TCRs, NK-cells activating or inhibitory signals are mediated by germline encoded receptors such as NKG2D, Ly49, or KIR (killer Ig-like receptor) and CD94–NKG2 heterodimers (94). Besides direct cytotoxicity using granzyme and perforine, triggered NK cells can also lead to the destruction of target cells by the stimulation of inflammatory cytokines production (95). Compared to T cells, the absence of TCRs strongly decreases the risk of GvHD, thus allowing the use of off-the-shelf available allogeneic CAR-NK cells. This could reduce the immense costs required for individual autologous products preparation, overcome the risk of malignant cells contamination of the final product, and also overcome other limits related to use of autologous cells of heavily pre-treated patient population. Umbilical cord derived CAR-NK cells have already been successfully used for treatment of CD19 malignancies (60) and several trials are active targeting this population using allogeneic CAR-NK cells (**NCT03056339, NCT04639739, NCT04887012, NCT04796675**). Active CAR-NK cells trial also target CD33/CLL1 for patients with acute myeloid leukemia (**NCT05215015**) or BCMA for multiple myeloma (**NCT05008536**). Use of this strategy in solid oncology is under clinical evaluation as well. Studies for patients with T-cell malignancies are commented below.

## Clinical Trials With CAR-Based Cellular Therapy in T-Cell Lymphomas

As of December 2021, 44 studies of CAR-T engineered cells were identified on Clinicaltrials.gov with the search “CAR-T” AND “T-cell lymphoma”, or “CAR-T” AND “lymphoblastic”. An additional five studies have been added after the screening of relevant literature (96, 97). Of the resulting 49 studies, more than 95% are conducted either in China (30, 61%) or in USA (17, 35%), including one study conducted both in China and USA and one study conducted in USA, Australia, and Canada. Only two studies are conducted in Europe. Forty-seven studies employed CAR-T cells, one study CAR-equipped myeloid cells, and one study cord-blood derived CAR-NK cells targeted against CD5 (**NCT05110742**).

The most common target is CD7 in 19 studies (39%), followed by CD30 (16 studies, 33%), CD4, and CD5 (5 studies or 10% each). Two studies direct CAR equipped cells against TRBC1 (**NCT04828174, NCT03590574**), one study against CD70 (**NCT04502446**), and one study against CD147 (**NCT05013372**). One study directs CAR-T cells simultaneously against CD30 and CCR4.

There are no Phase III studiesand 32 studies were annotated as Phase I, 11 as Phase I-II, 3 as Phase II, and in 3 studies Phase was not declared. Eight studies are not yet recruiting patients, 37 studies are actively recruiting, 1 study was completed, and the status of 3 studies is unknown.

All identified studies are comprehensively listed in **Supplementary Table 2**. Studies with at least partially published results are described in greater detail below and summarized in **Table 4**.

**Table 4:** Trials using CAR-T cells technology targeting T-cell malignancies with their corresponding results.

NCT identificator	Target antigen	Co-stimulation	Treated population and sample size	Lympho-depletion	Response	CRS (%)	ICANS (%)	Comment	Ref
NCT03081910	CD5	CD28	T-ALL (n=4), T-NHL (n=5)	Flu/Cy	ORR: 44%, CR: 33%	56	11		(98)
NCT04689659	CD7	NR	T-ALL (n=20)	Flu/Cy	ORR and CR: 90%	100	15	Allogeneic cells; KDEL CD7 expression blocker	(67)
NCT04572308	CD7	4-1BB	T-ALL (n=14)	Flu/Cy	ORR and CR: 93%	100	7	EGFR co-expression allowing CAR T-cells elimination with cetuximab	(99)
NCT04916860	CD7	4-1BB	T-LBL (n=8)	Flu/Cy	ORR: 75%, CR: 63%	100	13	EGFR co-expression allowing CAR T-cells elimination with cetuximab	(100)
NCT04004637	CD7	NR	T-ALL/LBL (n=8)	Flu/Cy	CR: 88%	100	NR	CD7 PEBL	(101)
NCT04264078	CD7	NR	T-ALL (n=5)	NR	CR: 100%	100	0	allogeneic cells with CRISPR/Cas9 induced TCRα and CD7 disruption	(102)
NCT01316146	CD30	CD28	HL (n=7), ALCL (n=2)	None	HL: 29% CR, ALCL: 50% CR	0	0		(103)
NCT02690545NCT02917083	CD30	CD28	HL (n=41), EATL (n=1)	Ben, Ben/Flu, Flu/Cy	HL: ORR 72%, CR 59%	25	0		(104, 105)
NCT02259556	CD30	NR	HL (n=17), ALCL (n=1)	Flu/Cy	ORR: 39%, CR 0%	100	25	Repeated infusions	(106)
NCT02663297	CD30	CD28	HL (n=1), ALCL (n=1)	Ben/Flu	HL: 50% CR, ALCL: 0% ORR	NR	NR	Consolidation after ASCT	(107)

ALCL, anaplastic large cell lymphoma; ALL, acute lymphoblastic leukemia; ASCT, autologous stem cell transplant; Ben, bendamustine; CR, complete remission; CRS, cytokine release syndrome; Cy, cyclophosphamide; EATL, enteropathy-associated T-cell lymphoma; EGFR, epidermal growth factor receptor; Flu, fludarabine; HL, Hodgkin’s lymphoma; ICANS, Immune effector cell-associated neurotoxicity syndrome; KDEL, K = lysine, D = aspartic acid, E = glutamic acid, L = leucin; NHL, non-Hodgkin’s lymphoma; NR, not reported; ORR, overall response rate; PEBL, protein expression blocker.

We identified five studies with results published in full-text manuscript: four of these targeted T-cells against CD30 and one against CD7. Studies with CAR-T cells against CD30 frequently treated also or mostly patients with Hodgkin’s lymphoma but as adverse events in CAR-Ts in general are more product-specific than disease-specific, the safety data are most probably relevant for T-cell non-Hodgkin’s lymphomas as well. **NCT01316146** is a Phase I study of anti-CD30 CAR-T lymphocytes conducted in the USA, which included seven patients with Hodgkin’s lymphoma and two patients with anaplastic large cell lymphoma (ALCL) (103). Three dose levels from 2x10^7^/m^2^ to 2x10^8^/m^2^ were tested and patients received from 1-4 infusion of cell product. No lymphodepletion regimen was used and probably because of its absence, no CRS or neurotoxicity was observed and other toxicities were not significant. As is the case of treatment with brentuximab vedotin, also antiCD30 CAR-T treatment antiCD30 CAR-T treatment did not induce non-malignant T-cell aplasia. However, CAR-T persistence was only transient and shorter with each subsequent infusion. One of ALCL patients achieved complete remission which was maintained for 9 months. The same product was used in two single-center Phase I-II studies (**NCT02690545** and **NCT02917083**), which treated 41 patients with relapsed and refractory Hodgkin’s lymphoma. The aforementioned three dose levels were used for the dose escalation cohort and 2x10^8^/m^2^ cells were used for the expansion cohort. In contrast with the previous study, lymphodepletion with bendamustine, bendamustine with fludarabine, or fludarabine with cyclophosphamide was used. Most common toxicities grade 3 or higher were rash and neutropenia (48% of patients both), CRS grade 1 developed in 25% of patients and no neurotoxicity was observed (104). One of these studies (**NCT02917083**), treated non-Hodgkin’s lymphoma patients as well and a separate case report of a patient with enteropathy-associated T-cell lymphoma, who achieved durable remission after antiCD30 CAR-Ts, was published (105).

In **NCT02259556**, 17 patients with Hodgkin´s lymphoma and 1 patient with cutaneous ALCL were treated with antiCD30 CAR-T cells. The median age was 33 years but the ALCL patient was 77 years old. After lymphodepletion with fludarabine and cyclophosphamide, 13 patients received one and 5 patients two CAR-T treatments. One treatment consisted of daily infusions of escalating numbers of CAR-T cells over 3-5 consecutive days up to total dose of 1.1-2.1x10^7^ cells/kg. All patients had grade 1-2 symptoms consistent with cytokine release syndrome and two cases of possible neurotoxicity occurred. Nearly all patients had cytopenias. There were two cases of rash and one case of elevated liver function test and decrease in left ventricular function each. The ALCL patient achieved partial remission of his skin lesions (106).

The only full-text study with CAR-T cells against other than CD30 antigen, **NCT04689659,** employed donor-derived antiCD7 CAR-T cells for patients with relapsed/refractory T-acute lymphoblastic leukemia (67). The CAR construct was equipped with an endoplasmic reticulum retention signal sequence (KDEL) to enable intracellular retention of CD7 molecules, thus limiting fratricide killing. Fludarabine and cyclophosphamide were used for lymphodepletion and single dose of 1x10^6^ cells/kg was administered. Among 20 treated patients, 90% achieved complete remission, with seven patients proceeding to alloHSCT. After median follow-up 6.3 months, 15 patients maintained complete remission. CRS occurred in all patients, but grade 3-4 CRS was observed only in two of them. Only three cases of neurotoxicity grade 1 were observed. Most common grade 3-4 toxicities were neutropenia and lymphocytopenia (both in 100%), thrombocytopenia in 95%, and anemia in 80%. One fatal fungal pneumonia occurred.

Several other studies have their results published as abstracts and most of these also use CAR-T cells against CD7 or CD30. An exception is a **NCT03081910** Phase I study of autologous CD5-directed CAR-T for R/R T-cell leukemia and lymphoma. First results were presented on ASH meeting in 2019. Four patients with T-ALL and five patients with T-NHL were treated on dose levels 1-2 (1x10^7^ CAR-T/m^2^ and 5x10^7^ CAR-T/m^2^, respectively). Their median age was 62 years (range, 16-71) and a single infusion was given after fludarabine and cyclophosphamide lymphodepletion. Five CRS grade 1-2, one neurotoxicity grade 2, and two prolonged cytopenias were observed. However, no complete CD3 depletion occurred. Three patients (one ALL, one AITL, and one PTCL) achieved complete remission. CAR-T cells were observed in bone marrow, lymph nodes, and in one case also in cerebrospinal fluid. Though the intention of this study was to bridge patients by CAR-Ts to alloHSCT, two of these patients declined it and relapsed after six weeks and seven months post-infusion (98). An update of this study was presented at the same meeting two years later. Total of 14 patients were treated at that time, 9 of which had peripheral T-cell malignancies and were presented in this update. Three of them received the third dose level (1x10^8^ CAR-T cells/m^2^). Seven patients received one infusion of CAR-T cells and two received additional infusion after initial disease evaluation. No new safety issues were noted. Four of nine patients achieved responses and three of them proceeded to alloHSCT. Clinical responses were observed on all dose levels, however, manufacturing process most likely influenced efficacy of CAR-T cells. Specifically, CAR-Ts expanded for shorter period of time were associated with better responses. Shortening of expansion from 7 to 3-5 also resulted in an enrichment of minimally differentiated CCR7+ CD62L+ T-cells (27-fold mean increase in CD4+, 13-fold increase in CD8+; p=0.01) and a 3.5-fold increase in CD27+ CD8+ T-cells (p=0.04) (108).

**NCT04572308** is a Phase I study of antiCD7 CAR-T cells for relapsed/refractory T-ALL developed in SenglangBio company, Hebei, China. The CAR construct is equipped with truncated EGFR protein, which enables elimination of CAR-T cells with anti-EGFR antibody cetuximab in case of severe toxicity. After lymphodepletion with fludarabine and cyclophosphamide, 0.5x10^5^ to 2x10^6^ cells/kg are administered in a three-step escalation design. Data for the first fourteen patients (median age 17 years, range, 3-42 years) were reportedand 13/14 patients achieved MRD negative remission while 11 of them were subsequently allotransplanted after a median of 57 days. Nine transplanted patients are in continuing remission. One relapse among the three non-transplanted patients was observed. All patients had CRS, but Grade 3 CRS was observed in one patient only. Neurotoxicity grade 1 was observed in one patient (99). First results of related Phase I study of this product in patients with R/R T-cell lymphoblastic lymphoma (**NCT04916860**) were also published. Data were reported for first eight patients with median age 37 years (14, 24–56). Autologous (n = 7) or donor-derived (n = 1) cells were used as a starting material. After lymphodepletion with fludarabine and cyclophosphamide, single dose of cells from 5x10^5^ to 2x10^6^/kg was infused. From the safety point of view, the patient who received CAR-Ts manufactured from his previous alloHSCT donor succumbed to acute graft-versus-host disease post CAR-T infusion. Grade 1-2 CRS occurred in seven and grade 3 CRS in one patient. All patients with bone marrow blasts pre-infusion achieved MRD negative status in marrow and 5 of 7 patients with extramedullary involvement achieved remission. However, only 2 patients had bulky disease before infusion and of these, only 1 achieved partial remission. No conclusions about long-term safety or efficacy can be drawn, as 6 of 8 patients received an allograft shortly after CAR-T cells (109). The *in vivo* kinetics of antiCD7 CAR-T product was compared to antiCD19 CAR-T cells also developed by SenglangBio. While antiCD19 CAR-Ts expanded significantly by day 7, peak numbers appeared on day 10, and drops to barely detectable levels were observed on day 21, antiCD7 CAR-T expanded by day 10 with peak at day 21 and were still detectable in significant numbers at day 30. Interestingly, this also corresponded with different profiles of cytokine release syndrome: After antiCD19 CAR-T infusion, CRS occurred at days 7-14, while two peaks of fever were noted at the first 3 days and then again day 10-21 after infusion of antiCD7 CAR-Ts (100).

Another Phase 1 antiCD7 CAR-T study for patients with T-ALL/LBL or ETP-ALL is **NCT04004637**. In addition to CD7-CAR, T cells were also co-transduced with CD7 protein expression blocker (PEBL) to avoid fratricide. 78% of attempted manufacturings were successful. Leukapheretic material used for successful productions had lower proportion of CD3+ cells and higher CD4+/CD8+ ratio (101). Patients received single dose of 1x10^6^-2x10^6^ CAR-T cells/kg after fludarabine and cyclophosphamide conditioning. Results for the first eight patients were reported. As in other studies with antiCD7 products, CRS was seen in all patients but was mild (grade 1-2). No other safety problems were reported. The response rate was 100% at one month and 75% at three months. Most patients ultimately progressed, but at least two of them enjoyed survival for more than 12 months after infusion (110).

**NCT04264078** is a Phase I study of allogeneic antiCD7 CAR-T cells (TruUCART™ CG027) with genomic disruption of TCRa and CD7 by CRISPR/Cas9 system to avoid GvHD and fratricide. Results for first five patients with R/R T-ALL were presented at the 2020 Meeting of American Association for Cancer Research (102). After lymphodepleting therapy, patients received 0.6x10^7^ to 1.5x10^7^ CG027 cells/kg. Toxicity was quite significant with all patients having grade 3-4 CRS. However, no neurotoxicity or GvHD was observed. All patients achieved MRD negative complete remission, which was maintained in four of them after a short follow-up (28-161 days). No patient received alloHSCT. Long-term results of the study are pending.

Regarding antiCD30 CAR-T cells, the first results of two Phase 1-2 studies from Lineberger Comprehensive Cancer Center were recently presented at the ASH meeting. In contrast to most other antiCD30 CAR-T studies, they included pediatric patients as well. Five patients, 9-17 years old (3 Hodgkin´s lymphomas, 2 ALCL), were treated with 1x10^8^/m^2^ cells in a “post-lymphodepletion” study after conditioning with bendamustine and fludarabine (**NCT02590545**). Two patients developed CRS and/or macrophage activation syndrome (MAS) grade 2, however, in one of them concomitant pneumonia was diagnosed and in the second case, the MAS was later attributed to progressive disease. One of the ALCL patients achieved complete remission. Two patients (1 Hodgkin´s lymphoma, 1 ALCL) were given 2x10^7^/m^2^ CAR-T cells as a consolidation after high-dose therapy and ASCT (**NCT02663297**). While the patient with Hodgkin´s lymphoma remained in complete remission, the ALCL patient progressed (107).

One of the obstacles for using allogeneic cells is their potential to cause severe graft-versus-host disease (109). Also, they may be the target of host immune system attack. To override problems with CRISPR/Cas9 or TALEN editing, Epstein-Barr Virus-specific T cells (EBVSTs) were used as a platform for CAR-T development, since they are virus specific rather than allospecific. CD30 CAR was introduced in these cells. CD30.CAR-EBVSTs resisted fratricide but were able to eliminate CD30-positive tumor cells. A bank of partially matched clinical grade CD30.CAR were developed for planned Phase 1 clinical trial (**NCT04288726**) (111). Results of first eight patients treated in this study were subsequently presented (112). Patients received lymphodepletion with fludarabine and cyclophosphamide. In subsequent cohorts, patients received from 4x10^7^ to 4x10^8^ CD30.CAR EBVSTs. There was no CRS and no graft-versus-host disease. An overall response rate was 71% (two complete and three partial remissions). However, histological diagnoses of these patients were not reported and the CAR-T cells were present in circulation only for one week and they did not expand. This seems to be a very safe treatment approach, but long-term results are difficult to predict. It was also concluded that CAR-Ts targeted to other antigens may be developed on the basis of the EBVST platform.

CCR4 is a chemokine receptor for CCL17. Its ligand is expressed on malignant cells of Hodgkin´s lymphoma and produces inhibitory barrier to cytotoxic T cells. Also, it mediates homing of T lymphocytes to skin (113). **NCT03602157** is a Phase I antiCD30 CAR-T cells co-expressing CCR4 for treatment of patients with Hodgkin´s lymphoma and CD30+ cutaneous T-cell lymphoma (CTCL). Lymphodepletion regimen consists of bendamustine and fludarabine and dose levels from 2x10^7^/m^2^ to 1x10^8^/m^2^ are employed. To date, results for the first 12 patients were reported, 2 of which had CTCL (114). Though responses were seen in all HL patients, one of patients with CTCL had stable disease only and the other one had progressive disease. Only three of 12 patients had CRS grade 1-2.

Despite the fact that many more studies were registered, they have no data available. Even though several of them employ a very attractive design for CAR-based therapies, the preclinical data are very limited. The potential of antiCD4 (**NCT04973527**, **NCT04712864**, **NCT04219319**, **NCT04162340** and **NCT 03829540**), antiTRBC1 (**NCT04828174** and **NCT03590574**, antiCD70 (**NCT04502446**) and antiCD147 (**NCT05013372**) CAR-T cells remains, so far, unknown, as well as the potential of NK cells (**NCT02742727**) and myeloid cells (**NCT05138458**) equipped with chimeric antigen receptor.

## Conclusions

Strategies using engineered CAR-cells have shown promising results in treating patients with B-cell malignancies and the field is growing rapidly. The use of CAR-T cells to treat T-cell malignancies is, however, complicated due to risk of fratricide, early exhaustion due to presence of target antigen on CAR-T cells, and the risk of transduction of malignant cells. There are various strategies to overcome these pitfalls, some of which have already been evaluated in clinical trials. Current developments allow modifications regarding the CAR structure, superficial antigens expression, as well as immune cells choice. Thus, we believe that CAR-based strategies are promising and will improve treatment options for the population in need, including the patients with T-cell malignancies.

## Author Contributions

KP and CS wrote the chapter “T-cell non-Hodgkin lymphomas”. KP draw the **Figure 1**. PO wrote the chapter “Basic concepts and challenges in chimeric antigen receptor-based therapy of T-cell malignancies”. RP wrote the Abstract and the chapter “Clinical trials with CAR-based cellular therapy in T-cell lymphomas”. All authors reviewed and approved the final manuscript.

## Funding

This publication was supported by a grant from Czech Ministry of Education, Youth and Sports OPVVV CZ.02.1.01/0.0/0.0/16_025/0007428.

## Conflict of Interest

The authors declare that the research was conducted in the absence of any commercial or financial relationships that could be construed as a potential conflict of interest.

## Publisher’s Note

All claims expressed in this article are solely those of the authors and do not necessarily represent those of their affiliated organizations, or those of the publisher, the editors and the reviewers. Any product that may be evaluated in this article, or claim that may be made by its manufacturer, is not guaranteed or endorsed by the publisher.
