# Supplementary material for: Chimeric Antigen Receptor Based Cellular Therapy for Treatment Of T-Cell Malignancies
Source: Front Oncol. 2022 May 6;12:876758. doi: 10.3389/fonc.2022.876758 (PMC9121778; doi:10.3389/fonc.2022.876758)
Supplement: Supplementary file 1 [file DataSheet_1.docx]

**Supplementary Table 1: Reported efficacies of selected targeted agents for R/R T-cell lymphomas treatment**

| **Drug** | **drug target** | **Target population** | **ORR %** | **PFS/DOR (months)** | **Trial identificator/ reference** |
| --- | --- | --- | --- | --- | --- |
| vorinostat | class I/II HDACi | R/R CTCL | 5 | 3.1 (PFS) | NCT01728805 |
|  |  |  | 30 | 6.6 (DOR) | NCT00091559 |
| romidepsin | class I HDACi | R/R PTCL | 25 | 4.0 (PFS) /25.0 (DOR) | NCT00426764 |
|  |  | R/R PTCL NOS and AITL | 42 | 2.5 (PFS) | (27) |
| chidamide | class I, II, III HDACi | R/R PTCL | 39 | 4.6 (PFS) | (37) |
| panobinostat | panHDACi | CTCL | 17 | 4.2 (PFS) | [NCT00425555](http://clinicaltrials.gov/show/NCT00425555) |
| pralatrexate | DHFR, TS | R/R PTCL | 29 | 3.5 (PFS) | NCT00364923 |
| mogamulizumab | CCR4 | R/R PTCL and CTCL | 35 | 3.0 (PFS) | NCT01192984 |
| alemtuzumab | CD52 | R/R PTCL | 36 |  | (40) |
| duvelisib | PI3K-δ,γ | R/R PTCL | 35-54 | 3.8 (PFS)/8.3 (DOR) | NCT03372057 |
| tenalisib | PI3K δ/γ | R/R PTCL and CTCL | 46 | 4.9 (DOR) | NCT02567656 |
| lenalidomid | CRL4^CRBN^ E3 ubiquitin ligase | R/R PTCL | 22 | 2.5 (PFS)/3.6 (DOR) | NCT00655668 |

**Abbreviations:** AITL – aingioimmunoblastic T-cell lymphoma; CCR4 – C-C chemokine receptor type 4; CRBN – cereblon; CRL4 - Cullin–RING ubiquitin ligase complex 4; CTCL – cutaneous T-cell lymphoma; DHFR – dihydrofolate reductase; DOR – duration of response; HDACi – histone deacetylase inhibitor; NOS – not otherwise specified; ORR – overall response rate; PI3K – phopshoinositide-3-kinase; PTCL – peripheral T-cell lymphoma; PFS – progression free survival; R/R – relapsed or refractory; TS – thymidylate synthase

**Supplementary Table 2: Clinical Trials of CAR-based cell therapy in T-cell malignancies**

| **NCT** | **Name** | **Country** | **Disease** | **Target** | **Phase** | **Auto/ allo** | **Age** | **Planned number of patients** | **Status** |
| --- | --- | --- | --- | --- | --- | --- | --- | --- | --- |
| **NCT04973527** | LCAR-T2C CAR-T Cells in Relapsed or Refractory CD4+ T Lymphocyte Tumor | China | T-ALL/T-LBL/ T-NHL | CD4 | I | Auto | 18-65 | 32 | Recruiting |
| **NCT04712864** | Study of CD4-Targeted Chimeric Antigen Receptor T-Cells (CD4- CAR-T) in Subjects With Relapsed or Refractory T-Cell Lymphoma | USA | PTCL/CTCL | CD4 | I | ??? | 18+ | 50 | Recruiting |
| **NCT04219319** | LCAR-T2C CAR-T Cells in Relapsed or Refractory CD4+ Tcell Lymphoma | China | T-NHL/T-ALL | CD4 | I | Auto | 18-75 | 32 | Recruiting |
| **NCT04162340** | CD4-specific CAR T Cells (CD4 CAR T Cells) for Relapsed/Refractory T Cell Malignancies | China | T-cell lymphoma | CD4 | I | ?? | 18+ | 12 | Recruiting |
| **NCT03829540** | CD4CAR for CD4+ Leukemia and Lymphoma | USA | T-ALL/T-LBL/ T-NHL | CD4 | I | Auto | 18+ | 20 | Not yet recruiting |
| **NCT05138458** | A Study of MT-101 in Subjects With CD5+ Relapsed/Refractory PTCL | USA | PTCL | CD5 | I/II | Allo?? | 18+ | 40 | Recruiting |
| **NCT04767308** | Safety and Efficacy of CT125A Cells for Treatment of Relapsed/Refractory CD5+ Hematopoietic Malignancies | China (?) | T-NHL/B-CLL/ B-NHL | CD5 | I | ??? | 18-70 | 18 | Not yet recruiting |
| **NCT04594135** | Anti-CD5 CAR T Cells for Relapsed/Refractory T Cell Malignancies | China | T-ALL/T-NHL | CD5 | I | ?? | 8+ | 20 | Recruiting |
| **NCT03081910** | Autologous T-Cells Expressing a Second Generation CAR for Treatment of T-Cell Malignancies Expressing CD5 Antigen | USA | T-ALL/T-LBL/ T-NHL/CTCL | CD5 | I | Auto | 0-75 | 42 | Recruiting |
| **NCT05032599** | Donor-Derived CD5 CAR T Cells in Subjects With Relapsed or Refractory T-Cell Acute Lymphoblastic Leukemia | China | T-ALL | CD5 | I | Allo (prior SCT or new donor) | 1-70 | 18 | Recruiting |

(continued)

| **NCT** | **Name** | **Country** | **Disease** | **Target** | **Phase** | **Auto/ allo** | **Age** | **Planned number of patients** | **Status** |
| --- | --- | --- | --- | --- | --- | --- | --- | --- | --- |
| **NCT05127135** | Safety and Efficacy of ThisCART7 in Patients With Refractory or Relapsed T Cell Malignancies | China | T-ALL/T-LBL/ T-NHL | CD7 | I | Allo | 3-70 | 30 | Recruiting |
| **NCT05059912** | CD7 CAR T-cell for R/R CD7+ T Cell Lymphoma | China | T-cell lymphoma | CD7 | II | ?? | 18+ | 20 | Recruiting |
| **NCT04984356** | A Phase 1/2 Study of the Safety and Efficacy of Anti-CD7 Allogeneic CAR-T Cells (WU-CART-007) in Patients With Relapsed or Refractory T-ALL/LBL | USA | T-ALL/T-LBL | CD7 | I/II | Allo | 12+ | 44 | Not yet recruiting |
| **NCT04934774** | Non-gene Edited Anti-CD7 CAR T Cells for Relapsed/Refractory T Cell Malignances | China | T-ALL/T-LBL/ T-NHL | CD7 | I | ?? | 18+ | 20 | Recruiting |
| **NCT04928105** | Senl-T7 CAR-T Cells for Treatment of Relapsed or Refractory CD7+ Lymphoma | China | T-NHL | CD7 | ?? | ?? | 2-70 | 100 | Recruiting |
| **NCT04916860** | Clinical Study of SenL-T7 CAR T Cells in the Treatment of Relapsed and Refractory CD7+ T-cell Lymphoblastic Leukemia or T-cell Lymphoblastic Lymphoma | China | T-ALL/T-LBL | CD7 | ?? | ?? | 2-70 | 100 | Recruiting |
| **NCT04860817** | A Study of Anti-CD7 CAR-T Cells in Pediatric and Young Adult Patients With Relapse and Refractory T-ALL/ T-LBL | China | T-ALL/T-LBL | CD7 | I | Allo (?) | 2-25 | 15 | Not yet recruiting |
| **NCT04840875** | Phase I Clinical Trial of Autologous CD7-CAR T Cells in the Treatment of High-risk Acute T-cell Leukemia / Lymphoma | China | T-ALL/T-LBL | CD7 | I | Auto | Up to 70 | 20 | Recruiting |
| **NCT04823091** | Autologous Cells Derived Anti-CD7 CAR-Engineered T Cells for T Lymphoid Malignancies | China | T-ALL/T-LBL/ T-NHL | CD7 | I | Auto | 18+ | 24 | Recruiting |

(continued)

| **NCT** | **Name** | **Country** | **Disease** | **Target** | **Phase** | **Auto/ allo** | **Age** | **Planned number of patients** | **Status** |
| --- | --- | --- | --- | --- | --- | --- | --- | --- | --- |
| **NCT04689659** | Multi-centers, Open-Label, Phase 2 Study to Evaluate the Efficacy and Safety of Donor-Derived CD7 CAR T Cells in Subjects With Relapsed or Refractory T-cell Leukemia/Lymphoma | China | T-ALL/T-LBL | CD7 | II | Donor | I.70 | 70 | Recruiting |
| **NCT04572308** | Cell Therapy for CD7 Positive T-cell Acute Lymphoblastic Leukemia and Lymphoblastic Lymphoma Using CD7-Specific CAR-T Cells | China | T-ALL/T-LBL | CD7 | I | Auto (?) | II.65 | 20 | Completed |
| **NCT04480788** | CD7-CART in the Treatment of r / r CD7 Positive HemolymphSystem Malignancies on Increasing Dose and Open Label Study | China | T-NHL/T-ALL/ T-LBL | CD7 | I | ?? | VII.70 | 9 | Recruiting |
| **NCT04264078** | Anti-CD7 U-CAR-T Cell Therapy for T/NK Cell Hematologic Malignancies | China | T-NHL/T-ALL/ T-LBL | CD7 | I | Allo | 18-70 | 30 | Recruiting |
| **NCT04033302** | Multi-CAR T Cell Therapy Targeting CD7-positive Malignancies | China | T-ALL/T-LBL/ AML/NKTCL | CD7 | I/II | Auto/Allo (previous donor) | 6 months-75 | 30 | Recruiting |
| **NCT04004637** | CD7 CAR-T Cells for Patients With R/R CD7+ NK/T Cell Lymphoma,T-lymphoblastic Lymphoma and Acute Lymphocytic Leukemia | China | T-ALL/T-LBL/ NK/T-NHL | CD7 | I | ?? | VII.70 | 10 | Recruiting |
| **NCT03690011** | Cell Therapy for High Risk T-Cell Malignancies Using CD7-Specific CAR Expressed On Autologous T Cells | USA | T-ALL/T-LBL/ T-NHL | CD7 | I | Auto | Up to 75 | 21 | Recruiting |
| **NCT02742727** | CAR-pNK Cell Immunotherapy in CD7 Positive Leukemia and Lymphoma | China | PTCL/ENKTL/ AML | CD7 | I/II | Allo | 18+ | 10 | Unknown |
| **NCT04620655** | RD13-01 for Patients With r/r CD7+ T-ALL/T-LBL | China | T-ALL/T-LBL | CD7 | ?? | Allo(?) | III.70 | 24 | Recruiting |

(continued)

| **NCT** | **Name** | **Country** | **Disease** | **Target** | **Phase** | **Auto/ allo** | **Age** | **Planned number of patients** | **Status** |
| --- | --- | --- | --- | --- | --- | --- | --- | --- | --- |
| **NCT04952584** | Allogeneic CD30 Chimeric Antigen Receptor Epstein-Barr Virus-Specific T Lymphocytes in Patients With Relapsed or Refractory CD30-Positive Lymphomas | USA | T-NHL/ENKTL/ HL | CD30 | I | Allo | 12-75 | 18 | Not yet recruiting |
| **NCT04653649** | CAR T-cells Against CD30 (HSP-CAR30) for Relapsed/Refractory Hodgkin and T-cell Lymphoma. | Spain | T-NHL/HL | CD30 | I/II | Auto | 18-70 | 30 | Recruiting |
| **NCT04526834** | Phase 1 Study of Autologous CD30.CAR-T in Relapsed or Refractory CD30 Positive Non-Hodgkin Lymphoma | USA | PTCL/ENKTL/ DLBCL/PMBL | CD30 | I | Auto | 18-75 | 21 | Recruiting |
| **NCT04288726** | Allogeneic CD30.CAR-EBVSTs in Patients With Relapsed or Refractory CD30-Positive Lymphomas | USA | T-NHL/HL | CD30 | I | Allo | 12-75 | 18 | Recruiting |
| **NCT04083495** | CD30 CAR for Relapsed/Refractory CD30+ T Cell Lymphoma | USA | PTCL | CD30 | II | Auto | 18-99 | 20 | Recruiting |
| **NCT04008394** | Anti-CD30 CAR-T Therapy in Patients With Refractory/Relapsed Lymphocyte Malignancies | China | PTCL/NKTCL/ HL | CD30 | I | Auto | 18-70 | 50 | Recruiting |
| **NCT03049449** | T Cells Expressing a Fully-Human Anti-CD30 Chimeric Antigen Receptor for Treating CD30-Expressing Lymphomas | USA | PTCL/ENKTL/ DLBCL/PMBL/ grey zone lymphoma | CD30 | I | Auto | 18-73 | 26 | Not yet recruiting |
| **NCT02917083** | CD30 CAR T Cells, Relapsed CD30 Expressing Lymphoma (RELY-30) | USA | T-NHL/HL | CD30 | I | Auto | 12-75 | 66 | Recruiting |
| **NCT02663297** | Administration of T Lymphocytes for Prevention of Relapse of Lymphomas | USA | T-NHL/ DLBCL/CTCL/HL | CD30 | I | Auto | 3+ | 18 | Recruiting |

(continued)

| **NCT** | **Name** | **Country** | **Disease** | **Target** | **Phase** | **Auto/ allo** | **Age** | **Planned number of patients** | **Status** |
| --- | --- | --- | --- | --- | --- | --- | --- | --- | --- |
| **NCT02274584** | CAR T Cells Targeting CD30 Positive Lymphomas (4SCAR30273) | USA/China | CD30+ lymphomas | CD30 | I/II | Auto | 18+ | 20 | Unknown |
| **NCT01316146** | Administration of T Lymphocytes for Hodgkin's Lymphoma and Non-Hodgkin's Lymphoma (CART CD30) | USA | CD30+ HL and NHL | CD30 | I | Auto | Children + adults | 10 | Not yet recruiting |
| **NCT02690545** | Study of CD30 CAR for Relapsed/Refractory CD30+ HL and CD30+ NHL | USA | CD30+HL and NHL | CD30 | I/II |  | 3+ | 40 | Recruiting |
| **NCT03383965** | CD30 Targeted CAR-T in Treating CD30-Expressing Lymphomas | China | HL, ALCL and other CD30+ malignancies | CD30 | I | Auto | II.80 | 20 | Recruiting |
| **NCT02958410** | A Clinical Research of CD30-Targeted CAR-T in Lymphocyte Malignancies | China | CD30-expressing lymphocyte malignancy | CD30 | I-II | ?? | 14-75 | 45 | Unknown |
| **NCT02259556** | CD30-directed Chimeric Antigen Receptor T (CART30) Therapy in Relapsed and Refractory CD30 Positive Lymphomas (CART30) | China | HL/NHL | CD30 | I-II | ?? | 16-80 | 30 | Recruiting |
| **NCT03602157** | Study of CAR-T Cells Expressing CD30 and CCR4 for r/r CD30+ HL and CTCL | USA | CTCL/HL/grey zone lymphoma | CD30/ CCR4 | I | Auto | 18+ | 59 | Recruiting |
| **NCT04502446** | A Safety and Efficacy Study Evaluating CTX130 in Subjects With Relapsed or Refractory T or B Cell Malignancies (COBALT-LYM) | USA/Australia/Canada | T-NHL/DLBCL | CD70 | I | Allo | 18+ | 45 | Recruiting |
| **NCT05013372** | CD147-CAR T Cells for Relapsed/Refractory T Cell Non-Hodgkin's Lymphoma | China | T-cell NHL | CD147 | I | ?? | 18-65 | 12 | Not yet recruiting |
| **NCT04828174** | Anti-TRBC1 CAR-T Cell Therapy in Patients With TRBC1 Positive T Cell Malignancies | China | T-ALL/T-LBL | TRBC1 | I | ?? | 18-70 | 9 | Recruiting |

(continued)

| **NCT** | **Name** | **Country** | **Disease** | **Target** | **Phase** | **Auto/ allo** | **Age** | **Planned number of patients** | **Status** |
| --- | --- | --- | --- | --- | --- | --- | --- | --- | --- |
| **NCT03590574** | Phase I/II Study Evaluating AUTO4 in Patients With TRBC1 Positive T Cell Lymphoma | Spain/United Kingdom | PTCL | TRBC1 | I-II | Auto | 18+ | 55 | Recruiting |

**Abbreviations:** AML – acute myeloid leukemia; B-NHL – B-cell non-Hodgkin´s lymphoma; CLL – chronic lymphocytic leukemia; CTCL – cutaneous T-cell lymphoma; DLBCL – diffuse large B-cell lymphoma; ENKTCL – extranodal killer/T-cell lymphoma; HL – Hodgkin´s lymphoma; NHL – non-Hodgkin´s lymphoma; NKTCL – natural killer and T-cell lymphoma; PMBL – primary mediastinal B-cell lymphoma; PTCL – peripheral T-cell lymphoma; T-ALL – T-cell acute lymphoblastic leukemia; T-LBL – T-cell lymphoblastic lymphoma; T-NHL – T-cell non-Hodgkin´s lymphoma
